# Supplementary material for: Highlighting the Biotechnological Potential of Deep Oceanic Crust Fungi through the Prism of Their Antimicrobial Activity
Source: Mar Drugs. 2021 Jul 24;19(8):411. doi: 10.3390/md19080411 (PMC8399467; doi:10.3390/md19080411)

Highlighting the biotechnological potential of deep oceanic crust fungi through the prism of their antimicrobial activity

Maxence Quemener[1], Marie Dayras[2], Nicolas Frotté[1], Stella Debaets[1], Christophe Lemeur[1], Georges Barbier[1], Virginia Edgcomb[3], Mohamed Mehiri[2], Gaëtan Burgaud[1*]

[1] Univ Brest, Laboratoire Universitaire de Biodiversité et Ecologie Microbienne, France

[2] Université Côte d'Azur, CNRS, Institut de Chimie de Nice, UMR 7272, Marine Natural Products Team, 06108, Nice, France.

[3] Departments of Geology and Geophysics and Biology, Woods Hole Oceanographic Institution, Woods Hole, MA 02543, USA

*****Correspondence: gaetan.burgaud@univ-brest.fr**;** Tel.: +33290915148

Supplementary Figure S1: Comparative analyses of the HPLC-PDA-ELSD chemical fingerprints of the crude organic extracts F1 of a) (UBOCC-A-118169 *Penicillium olsonii*), b) (UBOCC-A-119011 *Penicillium olsonii*) and c) (UBOCC-A-118156 *Aspergillus conicus*) dichloromethane-ethyl acetate extracts obtained with HPLC-DAD-ELSD (Macherey-Nagel propylphenyl-C18, 250 x 4.6 mm; 5 µm, gradient: CH_3CN + 0.1% formic acid (FA), H_2O + 0.1% FA, 10:90 to 100:0 in 30 mn).

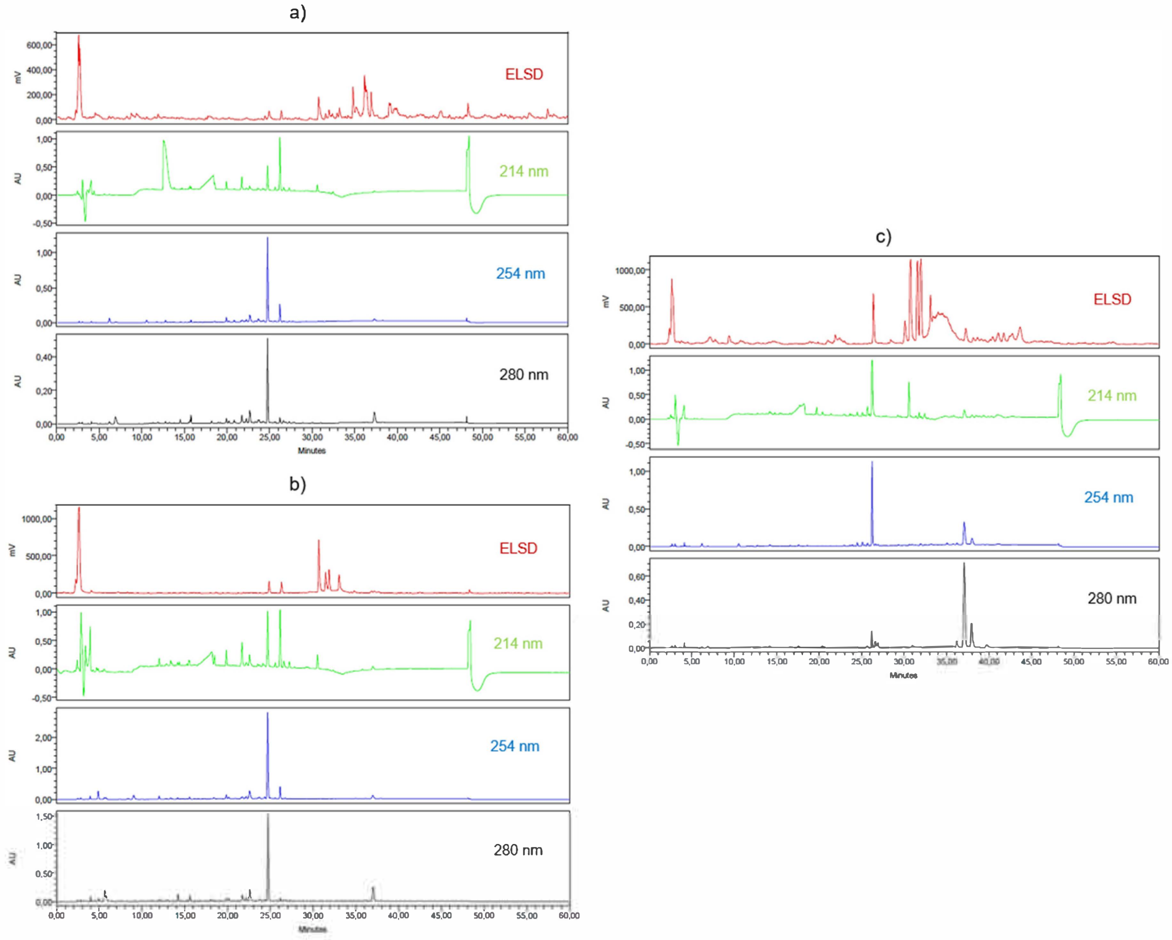

Supplement: Supplementary file 1 [file marinedrugs-19-00411-s001.zip › marinedrugs-1312746-supplementary.pdf]
